# Association mapping for canopy temperature under irrigated and rainfed conditions at 12 environments identified both known and novel genetic loci

**DOI:** 10.1002/tpg2.70272

**Published:** 2026-07-26

**Authors:** Siva K. Chamarthi, T. M. Shaikh, Felix B. Fritschi, Larry C. Purcell, Hussein Abdel‐Haleem, James R. Smith, Jason D. Gillman

**Affiliations:** ^1^ Division of Plant Science & Technology University of Missouri Columbia Missouri USA; ^2^ Department of Crop, Soil, and Environmental Sciences University of Arkansas Fayetteville Arkansas USA; ^3^ USDA‐ARS, United States Arid Land Agricultural Research Center Maricopa Arkansas USA; ^4^ USDA‐ARS Crop Genetics Research Unit Stoneville Mississippi USA; ^5^ USDA‐ARS Plant Genetic Research Unit University of Missouri Columbia Missouri USA

## Abstract

Soybean (*Glycine max* (L.) Merrill) is the most widely cultivated oilseed crop and faces significant yield challenges due to drought stress. Canopy temperature has been proposed as an effective proxy for evaluation of differences in genotypic responses to water limitation, as well as indirectly assessing transpiration and stomatal conductance. Cooler canopy temperatures are associated with higher transpiration and open stomata, whereas warmer canopy temperatures are associated with reduced transpiration and stomatal closure. This study evaluated a diverse group of 205 soybean maturity group IV genotypes across six irrigated and six rainfed environments. Genome‐wide association mapping identified 111 loci associated with normalized canopy temperature (nCT). Among these, only three loci overlapped with previously identified nCT loci, while 63 loci were aligned with other drought‐associated traits, and the remaining 45 loci were novel. Additionally, we identified 542 putative candidate genes in close proximity to significant single nucleotide polymorphisms, which have gene ontology annotations related to drought response mechanisms such as water transport, stomata regulation, response to temperature, and root development. Our findings were substantially consistent with previous studies on other drought tolerance traits, underscoring the interconnected nature of drought‐tolerance responses, as well as highlighting common genetic resources for breeding programs to develop more drought tolerant soybean cultivars. These confirmed genomic loci offer a valuable resource for enhancing soybean resilience to drought by pyramiding favorable alleles for nCT, thereby expediting breeding for cooler canopies.

## Plain Language Summary

The soybean crop significantly contributes to the US economy, with an estimated average annual impact of $124 billion. Drought is the greatest factor that negatively impacts soybean seed yields; on average, >20% of soybean growing areas are affected each year. Drought directly costs farmers income and indirectly costs the American public through crop insurance claims. Breeding drought‐tolerant soybean varieties is crucial to mitigate yield losses caused by drought. Toward this aim, we collected canopy temperature data, a high‐throughput trait related to drought tolerance, for 205 diverse soybean varieties across six irrigated and six rainfed environments. This rich dataset permitted detailed genetic analysis and identified new genetic sources of beneficial drought tolerance traits as well as regions of the genome containing drought tolerance genes. These results will accelerate ongoing breeding efforts to develop new soybean varieties more tolerant of drought stress.

**Abbreviations**
ANOVA: analysis of variance
BLUEs: best linear unbiased estimates
CT: canopy temperature
GS: genomic selection
GWAM: genome‐wide association mapping
IR: irrigated
MAS: marker‐assisted selection
MG: maturity group
nCT: normalized canopy temperature
QTL: quantitative trait loci
RF: rainfed
SNP: single nucleotide polymorphism
UAS: unmanned aerial systems
VPD: vapor pressure deficit
WUE: water use efficiency

## 1 INTRODUCTION

Soybean (*Glycine max* (L.) Merrill) is a crucial oilseed crop worldwide, essential for producing vegetable oil, protein feed for livestock, biodiesel, and a variety of soy‐based foods (American Soybean Association, 2025). In 2022, soybeans were grown on an estimated ∼134.2 million ha and ∼353.7 million metric tons were harvested worldwide (FAOSTAT, 2022). The soybean sector significantly contributes to the US economy, with an estimated average annual impact of $124 billion (LMC International, 2023). Drought stress is a significant threat to global agriculture, and limitations in soil moisture availability impair water uptake by plant roots and trigger various physiological, biochemical, and molecular changes in plants (Oguz et al., 2022; Salehi‐Lisar & Bakhshayeshan‐Agdam, 2020; Seleiman et al., 2021). These changes can decrease photosynthetic capacity (W. Wang et al., 2018), cause oxidative damage due to the accumulation of reactive oxygen species (Ahmad et al., 2014), generate metabolic disruptions (Kapoor et al., 2020), and alter gene expression (Gregorio Jorge et al., 2020). Large‐scale phenotypic impacts can include reduced plant growth, leaf wilting, altered root architecture, and ultimately lower crop yields (Bhat et al., 2020; Dubey et al., 2019; El Haddad et al., 2021), with effects ranging from minimal to resulting in no harvestable crop (Arya et al., 2021; Du et al., 2020; Xu et al., 2020).

### 1.1 Morphophysiological traits associated with drought tolerance

Breeding drought‐tolerant soybean varieties is crucial for reducing yield losses caused by water‐deficit conditions (Arya et al., 2021). Numerous morphophysiological traits associated with drought tolerance have been proposed and used to identify genotypes with drought tolerance potential, including a high ^13^C/^12^C carbon isotope ratio (Arifuzzaman et al., 2023; Bazzer, Kaler, King, et al., 2020; Bazzer, Kaler, Ray, et al., 2020; Chamarthi et al., 2023, 2024; Dhanapal, Ray, Singh, Hoyos‐Villegas, Smith, Purcell, King, Cregan, et al., 2015; Kaler, Dhanapal, et al., 2017; Steketee et al., 2020), the ratio of heavy to light oxygen isotopes (^18^O/^16^O) (Kaler, Dhanapal, et al., 2017), low canopy wilting (Abdel‐Haleem et al., 2012; Chamarthi et al., 2021; Charlson et al., 2009; Hwang et al., 2015, 2016; Kaler, Ray, et al., 2017; Li et al., 2024; Steketee et al., 2020), low canopy temperature (CT) (Bazzer & Purcell, 2020; Kaler, Ray, Schapaugh, Asebedo, et al., 2018), high N concentration and low ^15^N/^14^N ratios (Arifuzzaman et al., 2023; Bazzer, Ray, et al., 2020; Dhanapal, Ray, Singh, Hoyos‐Villegas, Smith, Purcell, King, & Fritschi, 2015; Steketee et al., 2019), high N derived from the atmosphere (Dhanapal, Ray, Singh, Hoyos‐Villegas, Smith, Purcell, King, & Fritschi, 2015) and C/N ratios (Arifuzzaman et al., 2023; Dhanapal, Ray, Singh, Hoyos‐Villegas, Smith, Purcell, King, & Fritschi, 2015), high canopy coverage (Kaler, Ray, Schapaugh, Davies, et al., 2018), leaf relative water content (Babu et al., 2003), rooting depth (Purcell & Specht, 2004), and root system architecture (Falk et al., 2020). Assessment of many of these and other physiological mechanisms contributing to drought tolerance can be laborious and slow, and the complex interactions between genotype, environment, and management make this endeavor even more challenging (Demicheli et al., 2023; Saleem et al., 2022). However, modern high‐throughput phenotyping platforms, such as unmanned aerial systems (UAS), enable more efficient, rapid, large‐scale genotype screening in the field, early detection of drought stress, and have the potential to indirectly report on water use efficiency (WUE) (Chen et al., 2014; Honsdorf et al., 2014; H. G. Jones, 2004; H. G. Jones et al., 2009; Thompson et al., 2020).

### 1.2 CT as a key indicator of drought responses

CT has been proposed as a crucial morphophysiological trait for assessing drought tolerance in crops (Lepekhov, 2022; Ninanya et al., 2021), including soybean (Bai & Purcell, 2018; Bazzer & Purcell, 2020; Kaler, Ray, Schapaugh, Asebedo, et al., 2018). CT has been utilized since the 1960s in field phenotyping to evaluate crop responses to drought (Fuchs & Tanner, 1966). CT hinges on the concept that leaf evaporation cools the canopy proportionally to the evapotranspiration rate. A cooler CT indicates higher rates of transpiration and stomatal conductance, whereas warmer CT suggests reduced transpiration and stomatal closure (H. G. Jones, 2004; H. G. Jones & Vaughan, 2010; Leinonen et al., 2006; Maes & Steppe, 2012), that is, transpiration rates are negatively correlated with CT due to evaporative cooling (Fukai & Mitchell, 2022). Therefore, CT is a valuable proxy for indirect evaluation of evapotranspiration, stomatal conductance, and their impacts on photosynthetic performance. Because isotopic traits (δ^1^ ^3^C, δ^1^ ^8^O, and δ^1^ ^5^N) are also shaped by variation in stomatal conductance, transpiration, and leaf internal CO_2_, they offer complementary insights into plant response to drought and WUE (Cabrera‐Bosquet et al., 2009; Farquhar et al., 1989).

Under optimal moisture conditions, a higher vapor pressure deficit (VPD) increases transpiration and leads to lower CT, provided stomatal conductance remains unchanged. However, reduced transpiration and stomatal closure under drought conditions lead to higher CT (Gates, 1968; H. Jones, 2010). Genotypes with cooler canopies under drought conditions tend to maintain higher transpiration rates, which can be linked with more efficient water use and a potential advantage in drought resilience (Reynolds et al., 2007, 2009). For instance, cooler CTs have been linked with higher grain yields in several crops, including soybean (Hou et al., 2019; Kumar et al., 2017), wheat (*Triticum aestivum*) (Aisawi et al., 2015; Balota et al., 2007), rice (*Oryza sativa*) (Fukai & Mitchell, 2022; Prince et al., 2015), sugarcane (*Saccharum officinarum*) (Basnayake et al., 2015; Chapman et al., 2014), chickpea (*Cicer arietinum*) (Purushothaman et al., 2015), and pearl millet (*Pennisetum americanum*) (Singh & Kanemasu, 1983) under water deficit conditions. Despite the ease of measurement, careful interpretation of CT data is critical. For instance, VPD‐responsive genotypes reduce stomatal conductance at high VPD under water‐replete conditions and thereby conserve soil moisture while also exhibiting higher CT (Monnens et al., 2023; Seversike et al., 2013). In simulation studies, restricted maximum evapotranspiration (slow canopy wilting) was predicted to result in increased yield in most field locations in >70% of years (Sinclair et al., 2010).

### 1.3 Genetic basis of CT and opportunities for crop improvement

CT is a complex quantitative trait influenced by genotype (G), environment (E), management (M), and their interactions (G × E × M) (Blum, 2011). The use of high‐throughput genotyping and phenotyping technologies, such as UAS, has facilitated the application of modern genomic tools like genome‐wide association mapping (GWAM) and genomic selection (GS) to investigate the genetic architecture of CT and other drought‐associated traits. Various single nucleotide polymorphism (SNP) datasets (hosted on https://soybase.org/), derived from the SoySNP50K iSelect SNP Beadchip from Illumina (Song et al., 2013, 2015), have been instrumental in the successful application of GWAM in soybean. Recently, we reported findings from a number of GWAM studies that identified significant genomic regions associated with other traits related to soybean drought tolerance, including canopy wilting (Chamarthi et al., 2021; Kaler, Ray, et al., 2017; Steketee et al., 2020; Ye et al., 2020), ^13^C/^12^C carbon isotope ratio as a surrogate measure for WUE (Arifuzzaman et al., 2023; Chamarthi et al., 2023, 2024; Dhanapal, Ray, Singh, Hoyos‐Villegas, Smith, Purcell, King, Cregan, et al., 2015; Kaler, Bazzer, et al., 2018; Kaler, Dhanapal, et al., 2017), and ^18^O/^16^O isotope ratio (Kaler, Dhanapal, et al., 2017) providing valuable insights for breeding more resilient soybean varieties.

### 1.4 Objectives

The current study builds upon an earlier study (Kaler, Ray, Schapaugh, Asebedo, et al., 2018), which assessed CT of diverse soybean genotypes across three environments. In the present study, we evaluated 205 diverse maturity group (MG) IV soybean genotypes in six irrigated (IR) and six rainfed (RF) environments, providing a more robust and comprehensive assessment of soybean CT responses to different environments.

The objectives of this study were threefold: First, to perform GWAM using a diverse panel of 205 MG IV soybean genotypes to identify genetic loci controlling CT; second, to investigate the relationship between CT and other drought tolerance traits, such as canopy wilting, ^13^C/^12^C, and ^18^O/^16^O; third, to identify the novel loci controlling CT.

## 2 MATERIALS AND METHODS

### 2.1 Genetic materials evaluated

A total of 205 MG IV soybean accessions were evaluated, including 199 diverse accessions as well as six controls: Plant Introduction (PI) 416937 (slow canopy wilting), PI 471938 (slow canopy wilting), A5959 (fast canopy wilting), 08705_16 (fast canopy wilting breeding line), LG11‐8169‐007F (MG IV elite breeding line), and Lee non‐nod (non‐nodulating check, PI 573285). LG11‐8169‐007F and Lee NN were planted in each of the 12 incomplete blocks in the field, serving as controls for symbiotic N fixation (though this was not relevant to the current study). The remaining 203 accessions were randomly assigned to incomplete blocks across three replications per treatment/site to facilitate nitrogen‐fixation‐related investigations. However, the data in this study were analyzed through a random complete block split‐plot IR/RF design. Of the 205 accessions, 99 were selected because they represent the majority of the genetic diversity present within a prior GWAM panel of 373 accessions (Kaler, Ray, Schapaugh, Asebedo, et al., 2018; Kaler, Dhanapal, et al., 2017). Another 100 accessions were chosen from the USDA‐GRIN collection (https://npgsweb.ars‐grin.gov/) based on predicted extremes for CT (Kaler, Ray, Schapaugh, Asebedo, et al., 2018), canopy wilting (Kaler, Ray, et al., 2017), ^13^C/^12^C carbon isotope ratio (Dhanapal, Ray, Singh, Hoyos‐Villegas, Smith, Purcell, King, Cregan, et al., 2015; Kaler, Dhanapal, et al., 2017), and symbiotic N fixation (Dhanapal, Ray, Singh, Hoyos‐Villegas, Smith, Purcell, King, Cregan, et al., 2015), as determined from high genomic estimated breeding values calculated from these previous association mapping studies.

### 2.2 Experimental site selection and management

The experiment was planned to have both IR and RF fields at each of four different research sites during the 2018 and 2019 cropping seasons. For clarity, we will refer to the combination of site, year, and treatment as an “environment.” The research sites included the Bradford Research Center in Columbia, MO (CO), the Maricopa Agricultural Center at the University of Arizona in Maricopa, AZ (MC), the Pine Tree Research Station in Arkansas (PT), and the Rohwer Research Station in Arkansas (RH); however, not all sites were included in both years or had both water availability treatments (due to excess precipitation, which precluded drought stress). In total, 12 designated site‐year–treatment combinations were included in this study: four from Columbia, MO (CO18IR, CO18RF, CO19IR, and CO19RF), four from Maricopa, AZ (MC18IR, MC18RF, MC19IR, and MC19RF), two from Pine Tree, AR (PT18IR and PT19RF), and two from Rohwer, AR (RH18IR and RH18RF).

Table S1 has comprehensive information on each site, including latitude, longitude, number of rows per plot, row length, row width, type of irrigation, planting date, CT measurement date, average maximum and minimum temperatures, total precipitation between planting and the CT measurement date, number of irrigations, irrigation dates, the approximate amount of water applied per irrigation, total irrigation amount, and cumulative soil moisture deficit. Cumulative soil moisture deficit was calculated by subtracting total precipitation and irrigation amount from potential evapotranspiration on a daily basis between emergence and the CT measurement date and then averaged (Purcell et al., 2007). Based on soil test results, we applied phosphorus and potassium at each site and used pesticides as needed. At the PT and RH sites, both IR and RF treatments received irrigation before the vegetative six‐trifoliolate (V6) stage.

### 2.3 CT phenotyping

CT measurements were taken under clear skies between 1200 and 1400 h at all site‐years. The measurements were taken only after the canopy was closed to prevent the measurements from being affected by the soil's heat signature. The soybean development stage selected for CT measurement was when most entries were at the beginning seed fill stage (R5), as in prior publications (Bai & Purcell, 2018; Bazzer & Purcell, 2020; Kaler, Ray, Schapaugh, Asebedo, et al., 2018).

At PT and RH, CT was measured via aerial infrared image analysis with a FLIR Tau 2 infrared camera mounted on a DJI Phantom 4 Pro drone (www.dji.com/phantom‐4‐pro). The camera, with a sensor size of 640 × 512 pixels and a 25‐mm focal length, recorded data with a sensitivity of 0.05°C and an accuracy of ±5%. The IR camera is not calibrated. That is, values of CT range from 0 (cool) to 255 (hot). Lower CT values correspond to cooler canopies (higher transpiration), whereas higher CT values indicate warmer canopies (reduced transpiration). Images were processed, and CT values were extracted using a previously published protocol (Bazzer & Purcell, 2020). Although thermal imaging was collected on multiple dates during the R5 stage, we used one representative date per site‐year images for final analysis to ensure consistency. Representative images of RF and IR field conditions are provided in Figure S1. Briefly, 5–10 images per plot were manually selected using a VLC video player, and the CT values were extracted using FieldAnalyzer software (www.turfanalyzer.com/field‐analyzer). Multiple CT values per plot were derived from the selected images, with final CT values representing the average across multiple drone‐collected images. At the CO location, CT was evaluated using a DJI Matrice 600 PRO UAV (DJI Inc.) with a Flir Duo Pro R, FLIR System, Inc. Images were processed using Agisoft Methashape Pro (Agisoft LLC). At the MC location, a high‐clearance tractor equipped with proximal sensors and imagers on a modified LeeAgra AvengerPro spray rig was used to collect CT data. Apogee SI‐131 infrared thermometer sensors were mounted on the platform and used to measure CT. Data were collected, stored, pre‐processed, and analyzed using in‐house pipelines (Thompson et al., 2020). Outliers were removed from raw data, and plot‐level data were calculated for CT using Python scripts.

To account for differences in measurement methods across site‐years, all CT data were normalized on a scale from 0 to 1 (Kaler, Ray, Schapaugh, Asebedo, et al., 2018). Normalized canopy temperature (nCT) was calculated using the following formula:

$$\mathrm{nCT}\;=\frac{Xi-\min\left(X\right)}{\max\;\left(X\right)-\min\left(X\right)}$$

where *Xi* represents the *i*th CT measurement in environment *X*, and min (*X*) and max (*X*) represent the minimum and maximum CT values for environment *X*, respectively.

### 2.4 Statistical analysis

Analysis of variance (ANOVA) was performed using the PROC GLIMMIX procedure of SAS 9.4 (SAS Institute Inc., 2023) to determine interactions among genotype, site‐year, and treatment. The statistical model for this analysis was *Y_ijkl_* = *μ* + *G_i_* + *S_j_* + *T_k_* + *GS_ij_* + *GT_ik_* + *ST_jk_* + *GST_ijk_* + *R_l_*_(_ *_jk_*_)_ + (residual error *ϵ_ijkl_*). In this model, fixed effects included *G_i_* is the effect of the *i*th genotype, *S_j_* is the effect of the *j*th site‐year, *T_k_* is the effect of the *k*th treatment, as well as all the fixed‐effect two‐way interactions (*GS_ij_*, *GT_ik_*, and *ST_jk_*) and the three‐way interaction *GST_ijk_*. The random effect is *R_l_*_(_ *_jk_*_)_ is the effect of the *l*th replicate nested in site‐year and treatment. The ESTIMATE statement was used to generate the best linear unbiased estimates (BLUEs) for each genotype in each site‐year–treatment combination using nCT. Raw nCT data are included in Table S2. The BLUEs employed in GWAM analysis are provided for all genotypes in Table S3.

To estimate the broad‐sense heritability (*H* ^2^), the PROC VARCOMP procedure of SAS 9.4 was used, applying the restricted maximum likelihood method to estimate variance components with all effects treated as random effects. Heritability was calculated using the following formula for each site‐year.

$$\;H^{2}=\frac{\sigma_{G}^{2}}{\left(\sigma_{G}^{2}+\left(\frac{\sigma_{\varepsilon }^{2}}{rk}\right)\right)}$$

where 𝜎^2^_𝐺_ is the genotypic variance, *k* is the number of site‐years, 𝜎^2^_𝜖_ is the residual variance, and *r* is the number of replications (Bernardo, 2002; Fehr, 1987). To assess consistency across environments, Pearson correlation coefficients were calculated using the “psych” package in R (Revelle, 2017).

### 2.5 Genotyping

We obtained marker data for 201 accessions from SoyBase (Glyma.w82.a1, www.soybase.org) using the Illumina Infinium SoySNP50K iSelect SNP Beadchip (Song et al., 2013, 2015). To ensure consistency in marker placement and alignment with previous studies, we used marker positions assigned for the Glyma.w82.a1 genome assembly. This process yielded 42,449 SNPs, which were subjected to quality control via TASSEL 5.0 (Bradbury et al., 2007). We removed SNPs with a minor allele frequency of ≤5%, a missing rate above 10%, and all monomorphic or heterozygous SNPs. After quality control, 34,680 SNPs remained. We imputed the minimal missing data in the filtered SNPs using the LD‐kNNi (Linkage Disequilibrium k‐Nearest Neighbor imputation) method (Money et al., 2015).

### 2.6 Genome‐wide association mapping

We conducted GWAM using the FarmCPU (Fixed and random model Circulating Probability Unification) option (Liu et al., 2016) in the GAPIT R package (J. Wang et al., 2022), a method known for its effectiveness in minimizing false positives and false negatives (Kaler et al., 2019; Liu et al., 2016). We applied a threshold value of −log10 *p* ≥ 3.5, which corresponds to a *p* value of ≤0.0003 and has been empirically validated as appropriate for soybean (Kaler & Purcell, 2019). Previous GWAM studies from our group have reported success using this threshold (Chamarthi et al., 2023, 2021, 2024; Kaler et al., 2020). We calculated the allelic effect of each significant SNP by determining the mean difference in nCT between the genotypes with major and minor alleles. A positive allelic effect indicates that the minor allele is associated with increased nCT, while a negative allelic effect indicates that the minor allele is associated with decreased nCT.

To identify overlaps between our study and previous ones focusing on drought‐related traits, we used the Bedtools Intersect Intervals Tool (Quinlan & Hall, 2010) within Galaxy (https://usegalaxy.org/), with an overlapping quantitative trait locus (QTL) region of ±175 kb (Chamarthi et al., 2023, 2021, 2024). We chose this window size because the average LD across all chromosomes decayed to an average of 175 kb in the euchromatic region, as previously described (Kaler et al., 2020). We considered SNPs that did not coincide with earlier studies as novel loci.

### 2.7 Candidate gene discovery

Regions around significant SNPs were investigated to identify soybean genes with annotations associated with abiotic stress responses (Glyma.w82.a1) via the Bedtools Intersect Intervals tool (Quinlan & Hall, 2010), including abscisic acid, jasmonic acid, water transport, root development, response to heat, temperature, and stomata based on gene ontology (GO) biological function (Sah et al., 2016; Schmutz et al., 2010). The output provided information on significant SNPs overlapping with putative candidate genes and their corresponding GO categories (Chamarthi et al., 2024). As before, an overlapping QTL region of ±175 kb was used.

## 3 RESULTS

### 3.1 Environmental variability

**FIGURE 1:**
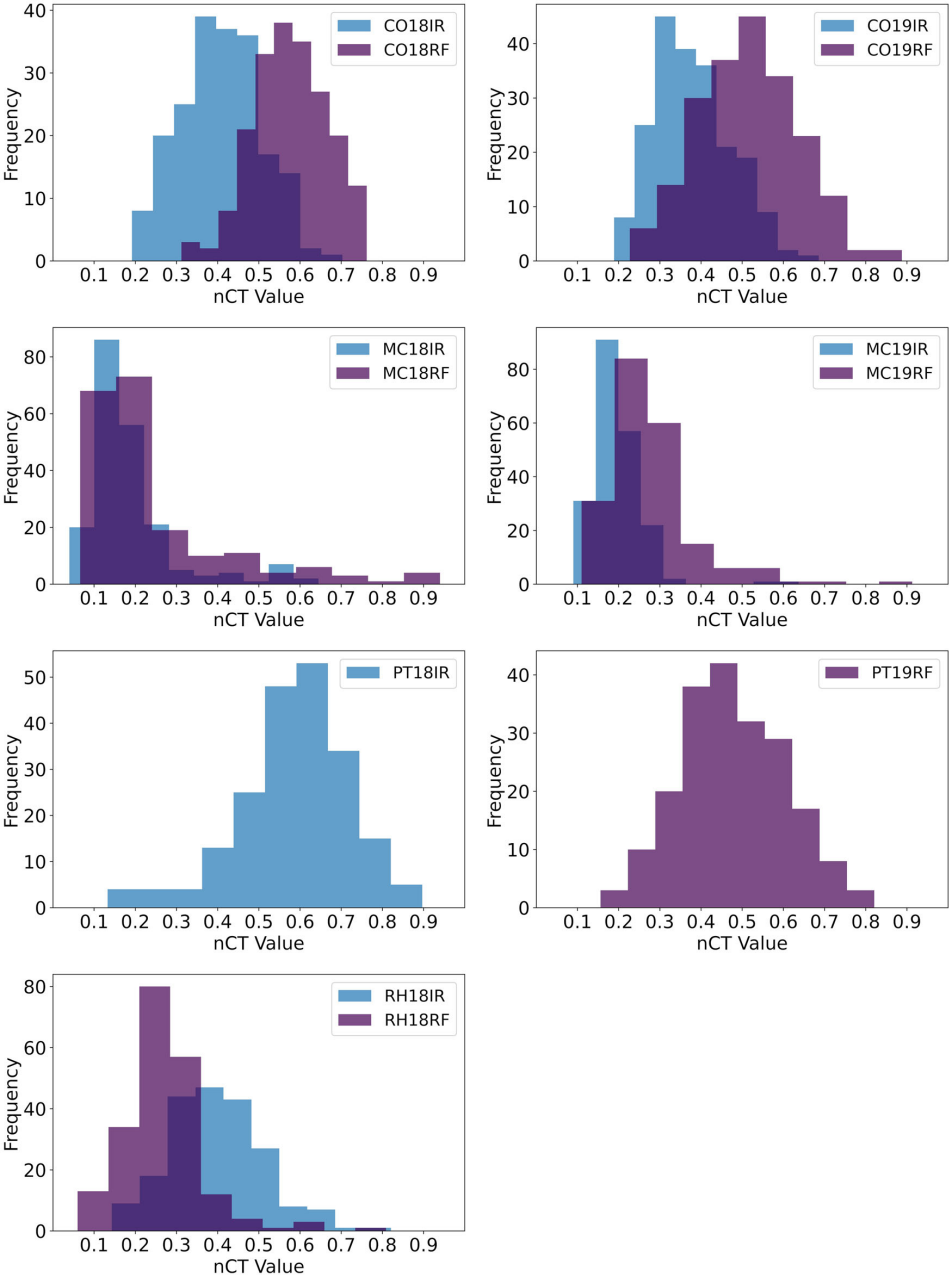
Frequency distribution of maturity group (MG) IV genotypes for normalized canopy temperature (nCT) best linear unbiased estimates (BLUEs) for six environments of irrigated and rainfed treatments: (a) Columbia 2018 IR&RF (CO18IR and CO18RF), (b) Columbia 2019 IR&RF (CO19IR and CO19RF), (c) Maricopa 2018 IR&RF (MC18IR and MC18RF), (d) Maricopa 2019 IR&RF (MC19IR and MC19RF), (e) Pine Tree 2018 IR (PT18IR), (f) Pine Tree 2019 RF (PT19RF), and (g) Rohwer 2018 IR&RF (RH18IR and RHRF).

We observed large differences in average maximum and minimum temperatures, as well as the total precipitation, among the 12 site‐year–treatment combinations during the period between emergence and the CT measurement dates (Table S1). The highest average maximum temperature (39°C) was recorded at MC18, while the lowest (28°C) was recorded at CO19. The highest average minimum temperature (23°C) was observed at RH18, while the lowest (17°C) was noted at CO19. The total precipitation from planting to the time of CT measurements was highest at PT19 at 376 mm and lowest in MC19 at 0 mm. The highest cumulative soil moisture deficit was observed at MC19RF at 427 mm, while the lowest was at PT19RF at −3 mm. Wide variations in nCT for both IR and RF site‐year–treatment combinations across different site‐year–treatment combinations were observed (Table 1). The minimum nCT range for the IR site‐year–treatment combinations was 0.57 (CO19IR), while the maximum was 0.76 (PT18IR). For the RF site‐year–treatment combinations, the minimum and maximum were 0.67 (CO18RF) and 0.87 (MC18RF), respectively (Table 1). The frequency distribution of nCT for each site‐year–treatment combination showed a broad range within and among IR and RF site‐year–treatment combinations (Figure 1).

**TABLE 1:** Descriptive statistics and broad sense heritability (*H* ^2^) of normalized canopy temperature (nCT) for 12 environments, Columbia (CO18 and 19), Maricopa (MC18 and 19), Pine Tree (PT18), and Rohwer (RH18) under irrigated (IR) and Columbia (CO18 and 19), Maricopa (MC18 and 19), Pine Tree (PT 19), and Rohwer (RH18) under rainfed (RF) treatments.

Environment	Mean	Standard deviation	Minimum	Maximum	*H* ^2^ (%)
CO18IR	0.41	0.10	0.19	0.70	3
CO19IR	0.38	0.09	0.19	0.69	20
MC18IR	0.19	0.11	0.04	0.64	47
MC19IR	0.20	0.06	0.09	0.64	64
PT18IR	0.59	0.13	0.13	0.90	64
RH18IR	0.40	0.11	0.14	0.82	57
CO18RF	0.58	0.09	0.31	0.76	23
CO19RF	0.52	0.12	0.23	0.89	26
MC18RF	0.25	0.18	0.07	0.94	82
MC19RF	0.28	0.10	0.11	0.91	74
PT19RF	0.48	0.13	0.16	0.82	59
RH18RF	0.27	0.10	0.06	0.81	71

### 3.2 Environmental interactions

**FIGURE 2:**
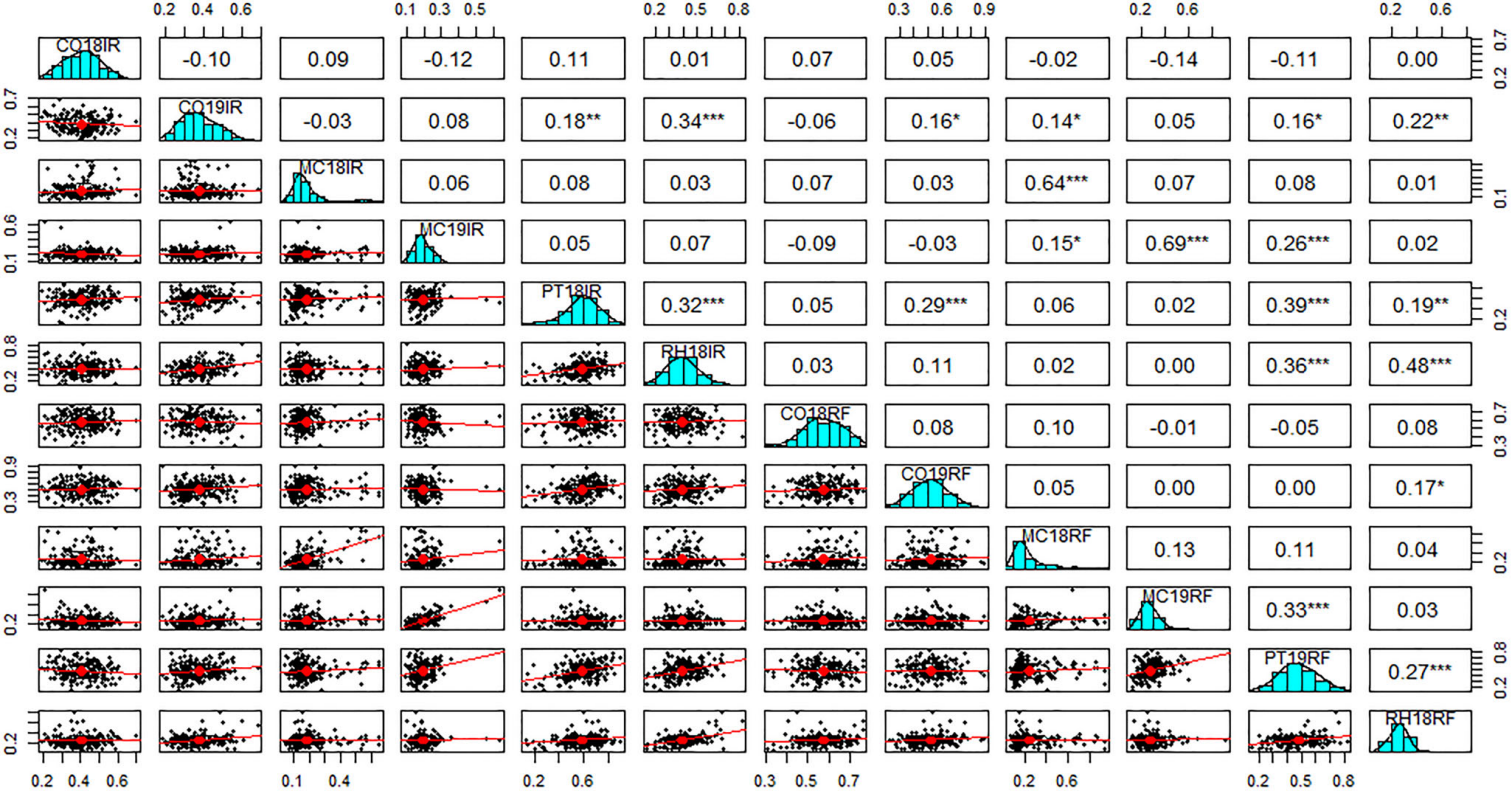
Correlations of normalized canopy temperature (nCT) (*n* = 205) for Columbia (CO18 and CO19), Maricopa (MC18 and 19), Pine Tree (PT18), and Rohwer (RH18) under irrigated (IR) and Columbia (CO18 and 19), Maricopa (MC18 and 19), Pine Tree (PT 19), and Rohwer (RH18) under rainfed (RF) treatments. Significance: *p ≤ 0.05, **p ≤ 0.01; ***p ≤ 0.001; ****p ≤ 0.0001.

ANOVA indicated that genotype, site‐year, site‐year–treatment combinations, and all their second‐order interactions were significant for nCT (*p* < 0.05). However, three‐way interactions were not significant (Table 2). Significant but weak positive correlations (*p* < 0.05) for nCT values between IR and RF site‐year–treatment combinations were observed for some comparisons (Figure 2). For the IR site‐year–treatment combinations, the strongest correlation was between RH18IR and CO19IR (0.34), while the weakest correlation was between PT18IR and CO19IR (0.18). For the RF site‐year–treatment combinations, the strongest correlation was between PT19RF and MC19RF (0.33), whereas the weakest was noted between RH18RF and CO19RF (0.17). When comparing IR and RF site‐year–treatment combinations, the strongest correlation was between MC19RF and MC19IR (0.69), while the weakest was between MC18RF and CO19IR (0.14) (Figure 2). Excluding CO18IR, for which *H^2^* was only 3%, *H* ^2^ for nCT ranged between 20% and 64% for IR site‐year–treatment combinations and 23% and 82% for RF site‐year–treatment combinations (Table 1).

**TABLE 2:** Analysis of variance across site‐year–treatment combinations for normalized canopy temperature (nCT).

Effect	Degree of Freedom	*F*‐statistic	*p*‐value	Significance
Genotype (G)	204	5.26	<0.0001	****
Site‐year (S)	6	17.23	<0.0001	****
Treatment (T)	1	5.21	0.0316	*
G × S	1215	2.38	<0.0001	****
G × T	204	1.34	0.0011	**
S × T	4	3.32	0.0266	*
G × S × T	804	1.08	0.0706	ns

Significance: ^ns^ *p* > 0.05; *p ≤ 0.05, **p ≤ 0.01; ***p ≤ 0.001; ****p ≤ 0.0001.

### 3.3 Genome‐wide association mapping

**FIGURE 3:**
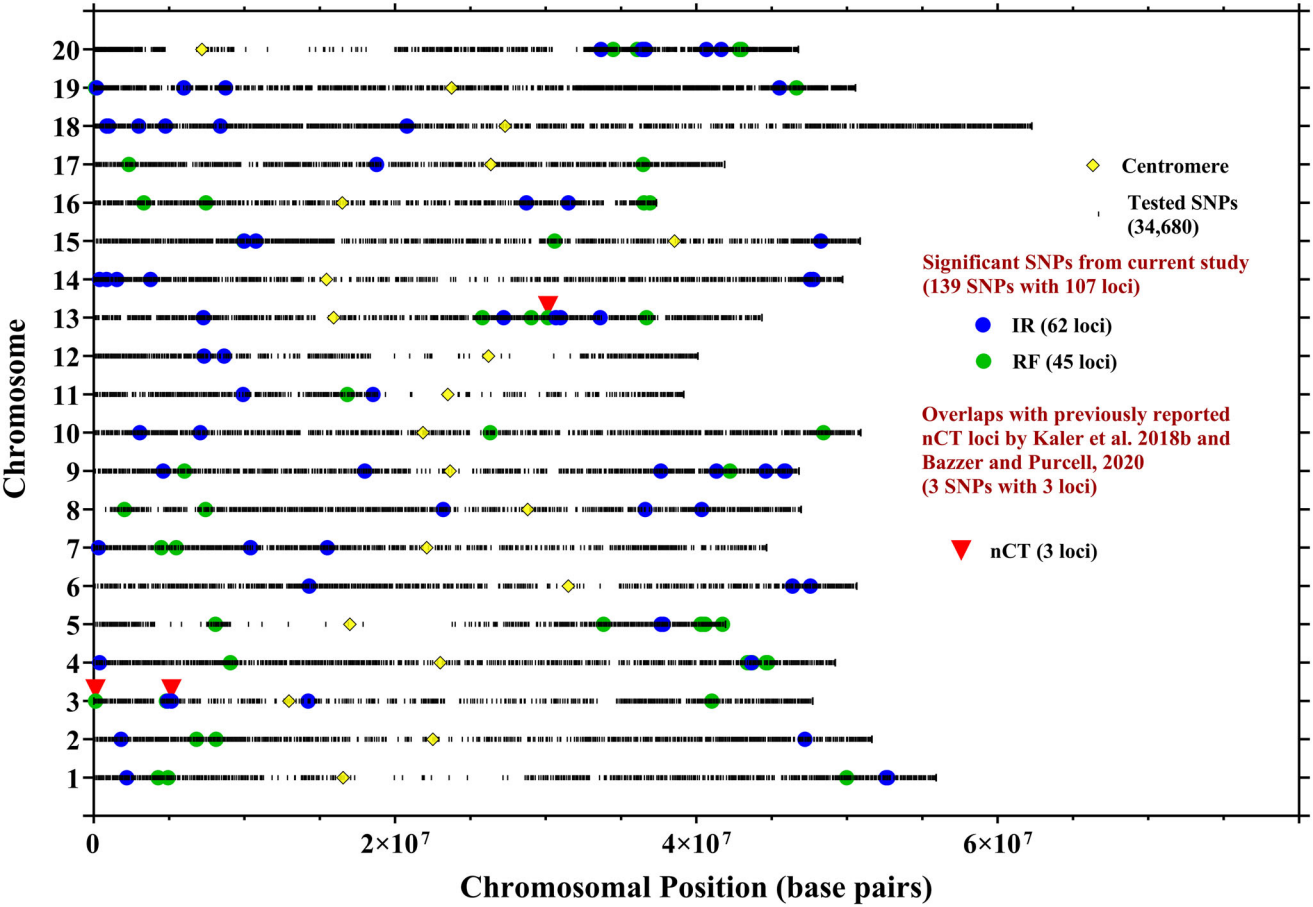
Locations of single nucleotide polymorphisms (SNPs) associated with normalized canopy temperature (nCT) from the current study compared with SNPs identified previously with nCT by Kaler, Ray, Schapaugh, Asebedo et al. (2018) and Bazzer and Purcell (2020). Details about the coincident SNPs are described in Table S4.

**FIGURE 4:**
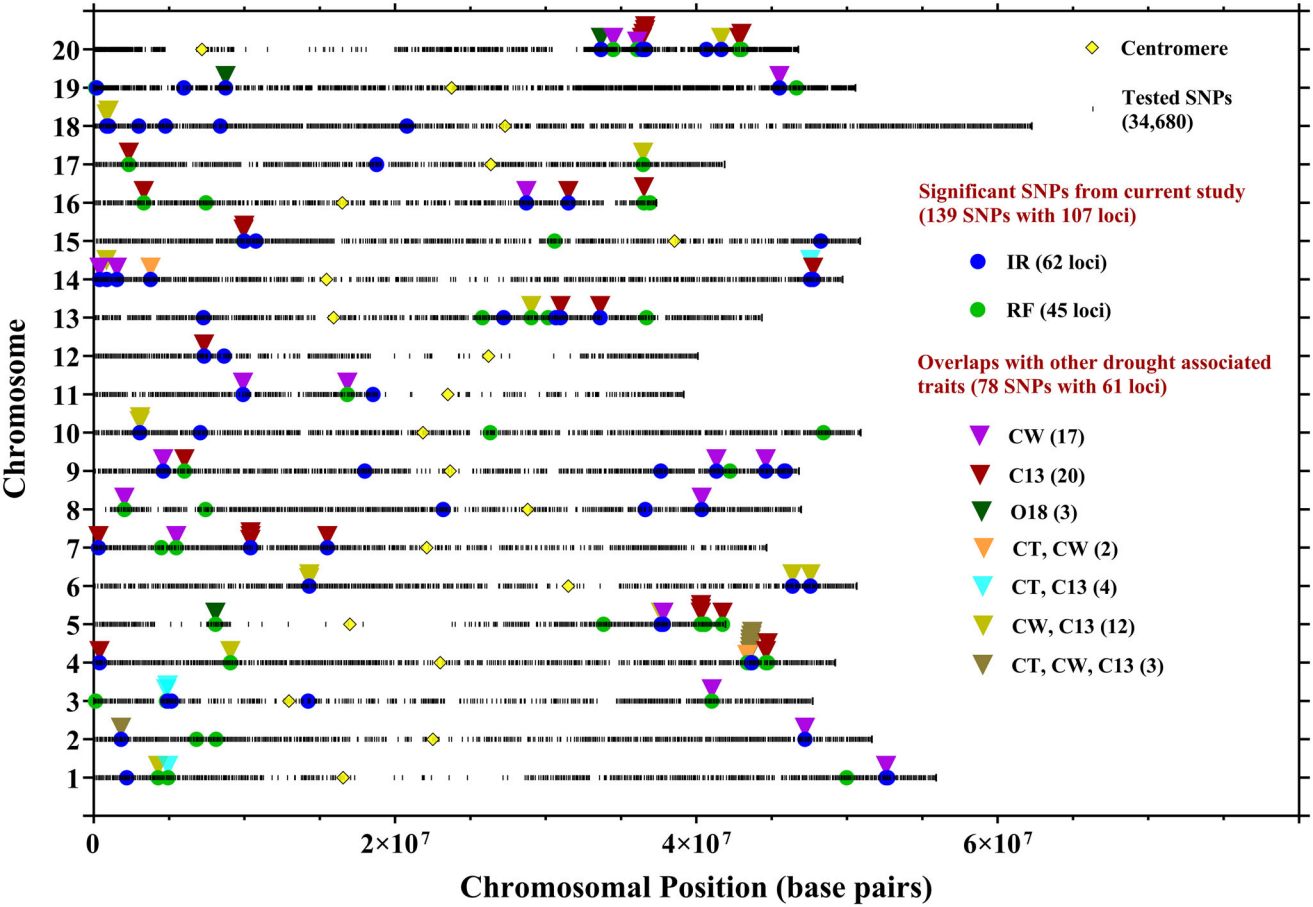
Locations of single nucleotide polymorphisms (SNPs) associated with normalized canopy temperature (nCT) from the current study compared with SNPs identified previously with other drought‐associated traits, such as canopy temperature (CT), canopy wilt (CW), carbon isotope ratio (C13), and oxygen isotope ratio (O18). Details about the coincident SNPs are described in Table S4.

A total of 139 significant SNPs, representing 111 loci, associated with nCT were identified by the GWAM study (Tables S4; Figures S2 and S3). Of these, 63 loci (83 SNPs) were mapped under IR conditions and 48 loci (56 SNPs) under RF conditions. Three loci, represented by SNPs ss715620247(Gm15), ss715633332(Gm19), and ss715636152(Gm19), were common in both conditions (Table S4, highlighted in green). Among these, we only identified three SNPs, representing three loci, that overlapped with nCT loci previously identified (Bazzer & Purcell, 2020; Kaler, Ray, Schapaugh, Asebedo, et al., 2018) (Figure 3). These SNPs ss715586763 (Gm03), ss715584381 (Gm03), and ss715615135 (Gm13) showed negative allelic effects ranging from −0.04 to −0.10 (Table S5.1), indicating that the major alleles are favorable for lowering CT. An additional 78 SNPs, representing 63 loci, coincided with loci previously reported for two other drought‐associated traits: ^13^C/^12^C and ^18^O/^16^O (Figure 4). Altogether, overlapping 81 SNPs (three for nCT and 78 for other drought‐associated traits) represented a total of 66 loci distributed across IR and RF site‐year–treatment combinations (Table S5.1). Forty‐seven SNPs representing 39 loci were identified at IR site‐year–treatment combinations, located on all chromosomes except Gm17, with allelic effects ranging from −0.17 to 0.15. Meanwhile, 34 SNPs representing 27 loci were found at RF site‐year–treatment combinations, on all chromosomes except Gm02, Gm06, Gm10, Gm12, Gm14, Gm18, and Gm19, with allelic effects ranging from −0.22 to 0.10 (Table S5.1).

Three SNPs, ss715611329 (Gm11 from IR), ss715588382 (Gm04 from RF), and ss715609383 (Gm11 from RF), share identical SNPs and positions previously associated with slower canopy wilting (Chamarthi et al., 2021; Kaler, Ray, et al., 2017). Additionally, one SNP, ss715588347 (Gm04 from RF), shares identical SNPs and positions linked to cooler CT (Kaler, Ray, et al., 2017). These four SNPs exhibit negative allelic effects of −0.07, −0.11, −0.08, and −0.09, respectively (Table S5.1, green highlighted). These regions almost certainly bear major alleles that were beneficial for slow wilting and cooler canopies, and their presence across multiple fields and experimental setups warrants further investigation.

Finally, of the total 139 SNPs we identified, 58 SNPs representing 45 loci were novel, meaning they have not previously been identified for drought‐related traits (Table S5.2). Of these, 36 SNPs representing 24 loci were from IR site‐year–treatment combinations with allelic effects ranging from −0.11 to 0.06, while 22 SNPs representing 21 loci were from RF site‐year–treatment combinations with effects ranging from −0.15 to 0.08.

### 3.4 Identification of putative candidate genes

A total of 135 of 139 significant SNPs were found to overlap with 542 putative candidate genes within a genomic region of ±175 kb. Among these, 360 were associated with SNPs coincident with previous studies (Table S6), while the remaining 182 were from novel SNPs (Table S7). Of the 135 overlapping SNPs, 13 were located directly within genes encoding proteins associated with plant stress responses (as opposed to being linked). Detailed information on the putative candidate genes and their predicted annotations for root development, water transport, heat stress, leaf vascular development, auxin, abscisic acid, gibberellic acid, jasmonic acid, leaf senescence, and stomatal movement regulation are located in Tables S6 and S7.

## 4 DISCUSSION

This study was carried out to generate a large phenotypic dataset of morpho‐physiological traits critical for drought tolerance, using a diverse panel of 205 soybean accessions grown in 12 site‐years, including six IR and six RF experiments. In our phenotypic effort, using both a drone‐based system and a tractor‐mounted sensor to measure CT gave us broader field coverage, but it also introduced clear differences since we used completely different thermal cameras across the three field locations. To account for that, we used a normalized strategy on location basis to harmonize the data. Our normalization step helped bring these measurements onto a comparable scale so they could be used together in the mapping analysis. Overall, this study enabled GWAM to identify both novel and previously identified genetic loci associated with CT, of which a substantial number overlapped with other morphophysiological traits associated with drought tolerance. We successfully confirmed three major genetic loci previously reported by Kaler, Ray, Schapaugh, Asebedo et al. (2018) and Bazzer and Purcell (2020) for nCT, along with an additional 61 major loci associated with other drought‐related traits. Furthermore, we discovered 43 new loci associated with nCT, expanding our knowledge of the genetic architecture underlying CT and providing new targets for breeding programs focused on enhancing drought tolerance in soybean.

### 4.1 Heritability and environmental interactions

Heritabilities calculated for each site‐year–treatment combination ranged from 3% to 82%, with a median of 58% (Table 1), indicating considerable genetic influence over this trait at some site‐years. However, exceptionally low heritability (3%) observed in CO18IR was most likely due to higher field heterogeneity, which increased environmental noise and reduced the proportion of variance explained by genotype. Substantial genetic control is encouraging for integration of new genetic tolerance sources for developing cultivars with consistent performance under varying environmental conditions. Correlation analysis of nCT values between different combinations of IR and RF site‐year–treatments revealed an absence of a significant relationship for most comparisons; significant positive relationships were only present for 19 of the 66 combinations explored and ranged from *r *= 0.14 to *r* = 0.69. Overall, these results demonstrate weak‐to‐moderate genetic effects across different site‐year–treatments (Figure 2). However, genotype–environment interactions were sizable and could pose challenges for selection (Hall et al., 2010). Previous studies have reported varying levels of heritability and correlations for CT in soybean (Bazzer & Purcell, 2020; Kaler, Ray, Schapaugh, Asebedo, et al., 2018) and wheat (Lopes & Reynolds, 2010; Mason et al., 2013), which is consistent with our findings.

### 4.2 Identification of genotypes with consistent nCT

During drought conditions, soybean genotypes with slower wilting exhibited cooler canopies, which were found to be positively correlated with higher grain yield (Bai & Purcell, 2018). Although our study did not measure seed yield, we were able to identify extreme genotypes with the lowest and highest nCT values for each site‐year–treatment (Table S8). Our comparative analysis with previous studies on CT (Kaler, Ray, Schapaugh, Asebedo, et al., 2018) and canopy wilting traits (Chamarthi et al., 2021; Kaler, Ray, et al., 2017) revealed overlapping genotype performance that allowed us to identify genotypes that consistently exhibited cooler, slow‐wilting canopies and those exhibiting warmer, fast‐wilting canopies (Table S9). For instance, genotypes PI407735 and PI432359 consistently showed cooler temperatures, while PI398298 consistently exhibited warmer temperatures, consistent with a previous report (Kaler, Ray, Schapaugh, Asebedo, et al., 2018). Furthermore, genotypes such as PI407735, PI407927B, PI548250, PI567447A, PI603457A, and PI603917 displayed cooler nCT values in our current study and were also found to wilt less in previous studies (Chamarthi et al., 2021; Kaler, Ray, et al., 2017). Conversely, genotypes PI 398601, PI 507408, and PI 594160 had relatively higher nCT values and wilted faster, consistent with the same studies. Our findings demonstrate strong consistency in genotype performance across experiments and methodologies, supporting their potential utility in breeding programs aimed at developing cultivars with cooler canopies and enhanced drought tolerance.

### 4.3 nCT genomic regions are also associated with other drought‐related traits

Association and linkage mapping studies have indicated that QTLs associated with different drought tolerance traits often co‐segregate. Examples include CT (Bazzer & Purcell, 2020; Kaler, Ray, Schapaugh, Asebedo, et al., 2018), canopy wilting (Abdel‐Haleem et al., 2012; Chamarthi et al., 2021; Charlson et al., 2009; Hwang et al., 2016; Kaler, Ray, et al., 2017; Li et al., 2024; Steketee et al., 2020), ^13^C/^12^C isotope ratio (Arifuzzaman et al., 2023; Bazzer, Kaler, King, et al., 2020; Bazzer, Kaler, Ray, et al., 2020; Chamarthi et al., 2023, 2024; Dhanapal, Ray, Singh, Hoyos‐Villegas, Smith, Purcell, King, Cregan, et al., 2015; Kaler, Dhanapal, et al., 2017; Steketee et al., 2019), and ^18^O/^16^O isotope ratio (Kaler, Dhanapal, et al., 2017).

**FIGURE 5:**
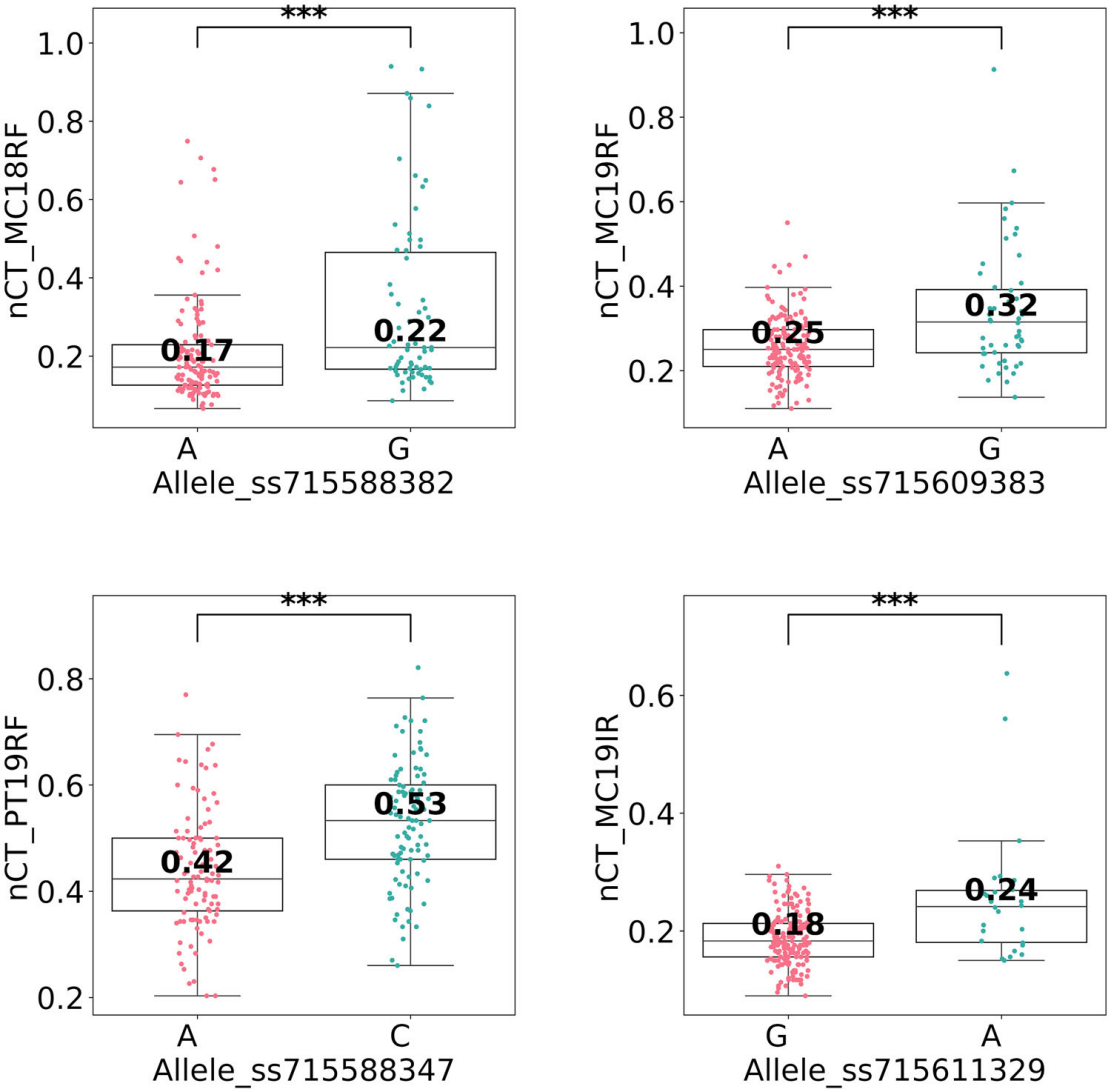
Box plot of consistent and high‐impact significant single nucleotide polymorphisms (SNPs) associated with normalized canopy temperature (nCT). SNP ss715588382 (top left) is located on Gm04, ss715609383 (top right) is located on Gm11, ss715588347 (bottom left) is on Gm04, and ss715611329 (bottom right) is located on Gm11. The *X*‐axis lists allele, while the *Y*‐axis displays the corresponding nCT genotypic mean for that specific environment. The median is the center line within the box plot. Asterisks (***) signify significant SNPs with a *p*‐value of ≤ 0.0003.

In our study, we identified 66 significant loci that overlap with genomic regions associated with drought‐related traits (Table S5.1), with three loci specifically linked to nCT (Bazzer & Purcell, 2020; Kaler, Ray, Schapaugh, Asebedo, et al., 2018) (Figure 3) and 63 associated with other drought‐related traits (Figure 4). Four notable SNPs for cooler nCT, ss715588347 (Gm04), ss715588382 (Gm04), ss715609383 (Gm11), and ss715611329 (Gm11) from the RF and IR site‐year–treatment combinations, share identical sequences and positions with those previously identified (Kaler, Ray, Schapaugh, Asebedo, et al., 2018), and for slower canopy wilting (Chamarthi et al., 2021; Kaler, Ray, et al., 2017). Their allelic effect for nCT ranged from ‐0.11 to ‐0.07 (Table S5.1; Figure 5). These SNPs were located near genes related to water transport, water deprivation, root development, response to heat, temperature, cold, and salt stress, stomatal complex morphogenesis, regulation of leaf formation, abscisic acid stimulus, and auxin stimulus, emphasizing their potential role in improving drought tolerance through genetic manipulation (Table S6). These consistent genomic regions highlight the potential for GS to improve drought tolerance in soybean by identifying genotypes with cooler canopies and delayed canopy wilting under drought stress.

### 4.4 Novel genetic loci associated with nCT

In addition to confirming previously identified nCT loci and collocation with loci of other drought‐related traits, our study identified 45 novel loci associated with nCT, several of which exhibited significant allelic effects. For instance, within the IR site‐year treatment combinations, SNP ss715609571 located on Gm11 demonstrated a minimum allelic effect of −0.11 (Table S5.2), accounting for 22% of the variation among genotypes for CO18IR. This SNP is situated near genes related to responses to heat, cold, salt stress, and water deprivation (Table S7). Similarly, SNP ss715629820 on Gm18 exhibited a maximum allelic effect of 0.06 (Table S5.2), accounting for 12% of the variation among genotypes for CO19IR. This SNP is near genes associated with root hair elongation, root morphogenesis, photosynthesis, cold response, and pathways related to jasmonic acid, salicylic acid, and auxin stimulation (Table S7).

From the RF site‐year–treatment combinations, SNP ss715633332 located on Gm19 exhibited a minimum allelic effect of −0.15 (Table S5.2), accounting for 17% of the variation among genotypes for MC18RF. This SNP is linked to genes associated with responses to oxidative stress, water deprivation, water transport, and signaling pathways involving abscisic acid, ethylene, and jasmonic acid (Table S7). Additionally, SNPs ss715591983 and ss715607917, located on Gm05 and Gm10, respectively, showed a maximum allelic effect of 0.08 (Table S5.2), accounting for 10% of the variation among genotypes for MC18 RF and MC19 RF. These SNPs are positioned near genes that respond to salt, heat, cold, and water deprivation and are involved in the jasmonic acid, abscisic acid, salicylic acid, and pentose phosphate shunt pathways (Table S7). Importantly, abscisic acid is a crucial hormone that regulates water status and stomatal aperture (Lim et al., 2015). These novel loci serve as potential targets for future functional characterization studies to elucidate their specific roles in regulating CT and drought response mechanisms in soybean.

### 4.5 Identification of candidate genes for nCT responses

We have identified 542 putative candidate genes within regions defined by significant coincident SNPs (Table S6) and novel SNPs (Table S7). Among these, several genes of particular interest were found near published genes listed in SoyBase (https://www.soybase.org/). For instance, SNP ss715579960 on Gm01 is located near *Glyma01g05170*, which belongs to the UDP‐galactose transporter protein family, and is associated with RF treatments (Table S6). Previous studies have shown that the overexpression of *AtGolS2* in genetically modified soybean plants increases galactinol transcript levels, leading to changes in carbohydrate metabolism that enhances drought tolerance and yield under both IR and drought conditions (Honna et al., 2016). Another significant SNP, ss715638471 on Gm20, is situated near the *Glyma20g34381*, which belongs to the auxin efflux carrier protein family and is also associated with RF treatments (Table S6). Prior research demonstrated that overexpressing the *OsPIN3t* gene improves drought tolerance in rice (Zhang et al., 2012).

### 4.6 Context of this study within recent scientific literature

Recent advances in the genetics of soybean drought response provide significant context for our nCT GWAS study. Burner et al. (2025) identified seven canopy wilting QTLs distributed across six chromosomes in a PI 603535, establishing a strong correlation between NDVI and GNDVI with canopy wilting (|*r*| = 0.42–0.44). The colocalization of vegetation index QTLs with canopy wilting loci highlights the complex and polygenic nature of drought tolerance. Menke et al. (2024) also reported that slow canopy wilting exhibits low heritability (*H* ^2^ = 0.29) and is governed by numerous minor effect QTLs that vary across environments. This shows how sensitive canopy‐level drought traits are to the environment and how useful GS can be for making plants more drought‐resistant. Additional GWAS studies have identified drought‐ and heat‐responsive loci pertinent to CT, encompassing those associated with canopy wilting, leaf senescence, stomatal regulation, and growth responses under short‐ and long‐duration drought (Saleem et al., 2022), as well as yield‐related drought traits (Li et al., 2023). Heat stress GWAS further demonstrates significant genotype by temperature interactions, revealing 37 significant marker trait associations detected under both optimal and heat stress conditions (Van der Laan et al., 2025), reinforcing that canopy‐level thermal traits are shaped by a complex, environment dependent genetic architecture. Collectively, these recent findings support the relevance of the SNPs and candidate genes identified in our study and emphasize the importance of integrating multi‐environment phenotyping with genomic approaches to dissect CT variation.

### 4.7 Potential for this study to improve soybean breeding for drought tolerance

The genomic loci and candidate genes identified in this study provide valuable resources for accelerating drought tolerance improvement in soybean breeding programs. The 1087 SNPs associated with nCT, including 43 novel loci and 61 loci overlapping with previously reported drought‐related traits, can be directly incorporated into marker‐assisted selection (MAS) to introgress favorable alleles associated with cooler canopies and improved transpiration efficiency. The 542 candidate genes linked to water transport, stomatal regulation, temperature response, and root development also offer strong targets for GS, where integrating nCT‐associated markers can enhance prediction accuracy for drought‐adaptive traits. The multi‐environment stability of several loci increases their utility for breeding under variable RF conditions, aligning with recent findings that canopy‐level drought traits benefit from genomic‐enabled selection strategies (Burner et al., 2025; Menke et al., 2024). Together, these results demonstrate that the loci identified in our study can be used to pyramid favorable alleles for cooler CTs, thereby expediting the development of drought‐resilient soybean cultivars (Li et al., 2023; Saleem et al., 2022; Van der Laan et al., 2025).

### 4.8 Future directions

Future research should prioritize studying the functional roles of identified putative candidate genes and SNPs and their integration into breeding pipelines. By understanding the genetic makeup of CT and its interaction with environmental factors, breeders can develop cultivars that can maintain cooler CTs under stress conditions, which have been associated with reduced yield losses due to water scarcity. Additionally, a detailed analysis of the identified genomic regions and physiological studies to identify causative mechanisms will be pivotal in advancing our understanding of drought tolerance in soybean.

## 5 CONCLUSIONS

This study provides a comprehensive analysis of the genetic architecture of CT and its significance in drought tolerance in soybean. Extensive evaluation of a diverse panel of 205 accessions showed strong environmental influence on CT and highlighted the importance of multi‐environment trials for rigorous drought research. High heritability for CT, supporting its value as a selection trait. Our GWAS study identified 111 significant loci associated with altercation in nCT, including previously reported regions, loci coincident with other drought‐related traits, and 45 novel loci near annotated genes involved in critical stress response pathways, for example, auxin signaling, abscisic acid regulation, water transport, or root development. Several robust SNPs on Gm04 and Gm11 (ss715588347, ss715588382, ss715609383, and ss715611329) were consistently associated with lower CT and delayed wilting, highlighting their potential for MAS and GS. Genotypes with stable low CT across environments, such as PI407735 and PI432359, also emerged as promising breeding materials. Future work should prioritize functional validation of key genotypes and candidate genes and the integration of both novel and known loci into breeding pipelines to advance drought‐resilient soybean development.

## AUTHOR CONTRIBUTIONS

**Siva K. Chamarthi**: Formal analysis; investigation; visualization; writing—original draft. **T. M. Shaikh**: Formal analysis; writing—review and editing. **Felix B. Fritschi**: Conceptualization; data curation; investigation; resources; supervision; writing—review and editing. **Larry C. Purcell**: Conceptualization; data curation; funding acquisition; investigation; project administration; supervision; writing—review and editing. **Hussein Abdel‐Haleem**: Data curation; writing—review and editing. **James R. Smith**: Data curation; writing—review and editing. **Jason D. Gillman**: Conceptualization; data curation; formal analysis; funding acquisition; investigation; project administration; supervision; writing—review and editing.

## CONFLICT OF INTEREST STATEMENT

The authors declare no conflicts of interest.

## Supporting information




**Table S1**: Details on environmental data for each field location.
**Table S2**: Normalized canopy temperature (nCT) raw data for each environment.
**Table S3**: nCT BLUE values for each environment of six irrigated and six rainfed treatments.
**Table S4**: Significant SNPs overall.
**Table S5.1 and S5.2**: Significant coincident and novel SNPs identified from the current study associated with nCT.
**Table S6**: List of 81 significant coincident SNPs associated with nCT and their potential candidate gene overlaps.
**Table S7**: List of 58 significant novel SNPs associated with nCT and their potential candidate gene overlaps.
**Table S8**: Top 10 cooler and warmer genotypes from each site‐year‐treatment combination.
**Table S9**: Comparative analysis of top and bottom 10 cooler and warmer genotypes in the current study with those from previous studies.
**Fig. S1**: Representative images of field conditions under rainfed and irrigated treatments; (A) Pine Tree Irrigated (PT18IR), B) Pine Tree Rainfed (PT19RF), (C) Rohwer Irrigated (RH18IR), and (D) Rohwer Rainfed (RH18RF).
**Fig. S2**: Manhattan plots of ‐Log10 (P) vs. chromosomal position of significant SNP associations of nCT for six environments under irrigated treatment.
**Fig. S3**: Manhattan plots of ‐Log10 (P) vs. chromosomal position of significant SNP associations of nCT for six environments under rainfed treatment.

## Data Availability

The authors affirm that all data necessary for confirming the conclusions of the article are present within the article, figures, tables, and supplementary files.

## References

[tpg270272-bib-0001] Abdel‐Haleem, H. , Carter, T. E., Jr. , Purcell, L. C. , King, C. A. , Ries, L. L. , Chen, P. , Schapaugh, W., Jr. , Sinclair, T. R. , & Boerma, H. R. (2012). Mapping of quantitative trait loci for canopy‐wilting trait in soybean (*Glycine max* L. Merr). Theoretical and Applied Genetics, 125(5), 837–846. 22566068 10.1007/s00122-012-1876-9

[tpg270272-bib-0002] Ahmad, P. , Jamsheed, S. , Hameed, A. , Rasool, S. , Sharma, I. , Azooz, M. M. , & Hasanuzzaman, M. (2014). Drought stress induced oxidative damage and antioxidants in plants. In P. Ahmad (Ed.), Oxidative damage to plants (pp. 345–367). Academic Press.

[tpg270272-bib-0003] Aisawi, K. A. B. , Reynolds, M. P. , Singh, R. P. , & Foulkes, M. J. (2015). The physiological basis of the genetic progress in yield potential of CIMMYT spring wheat cultivars from 1966 to 2009. Crop Science, 55(4), 1749–1764.

[tpg270272-bib-0004] American Soybean Association . (2025). Soy stats . http://soystats.com/

[tpg270272-bib-0005] Arifuzzaman, M. , Mamidi, S. , Sanz‐Saez, A. , Zakeri, H. , Scaboo, A. , & Fritschi, F. B. (2023). Identification of loci associated with water use efficiency and symbiotic nitrogen fixation in soybean. Frontiers in Plant Science, 14, 1271849. 38034552 10.3389/fpls.2023.1271849 PMC10687445

[tpg270272-bib-0006] Arya, H. , Singh, M. B. , & Bhalla, P. L. (2021). Towards developing drought‐smart soybeans. Frontiers in Plant Science, 12, 750664. 34691128 10.3389/fpls.2021.750664 PMC8526797

[tpg270272-bib-0007] Babu, R. C. , Nguyen, B. D. , Chamarerk, V. , Shanmugasundaram, P. , Chezhian, P. , Jeyaprakash, P. , Ganesh, S. K. , Palchamy, A. , Sadasivam, S. , Sarkarung, S. , Wade, L. J. , & Nguyen, H. T. (2003). Genetic analysis of drought resistance in rice by molecular markers. Crop Science, 43(4), 1457–1469.

[tpg270272-bib-0008] Bai, H. , & Purcell, L. (2018). Aerial canopy temperature differences between fast‐and slow‐wilting soya bean genotypes. Journal of Agronomy and Crop Science, 204(3), 243–251.

[tpg270272-bib-0009] Balota, M. , Payne, W. A. , Evett, S. R. , & Lazar, M. D. (2007). Canopy temperature depression sampling to assess grain yield and genotypic differentiation in winter wheat. Crop Science, 47(4), 1518–1529.

[tpg270272-bib-0010] Basnayake, J. , Jackson, P. , Inman‐Bamber, N. , & Lakshmanan, P. (2015). Sugarcane for water‐limited environments. Variation in stomatal conductance and its genetic correlation with crop productivity. Journal of experimental botany, 66(13), 3945–3958. 25948709 10.1093/jxb/erv194

[tpg270272-bib-0011] Bazzer, S. K. , Kaler, A. S. , King, C. A. , Ray, J. D. , Hwang, S. , & Purcell, L. C. (2020). Mapping and confirmation of quantitative trait loci (QTLs) associated with carbon isotope ratio (δ^13^C) in soybean. Crop Science, 60(5), 2479–2499.

[tpg270272-bib-0012] Bazzer, S. K. , Kaler, A. S. , Ray, J. D. , Smith, J. R. , Fritschi, F. B. , & Purcell, L. C. (2020). Identification of quantitative trait loci for carbon isotope ratio (δ13C) in a recombinant inbred population of soybean. Theoretical and Applied Genetics, 133(7), 2141–2155. 32296861 10.1007/s00122-020-03586-0

[tpg270272-bib-0013] Bazzer, S. K. , & Purcell, L. C. (2020). Identification of quantitative trait loci associated with canopy temperature in soybean. Scientific Reports, 10(1), Article 17604. 33077811 10.1038/s41598-020-74614-8 PMC7572360

[tpg270272-bib-0014] Bazzer, S. K. , Ray, J. D. , Smith, J. R. , Fritschi, F. B. , & Purcell, L. C. (2020). Mapping quantitative trait loci (QTL) for plant nitrogen isotope ratio (δ15N) in soybean. Euphytica, 216(12), Article 191.

[tpg270272-bib-0015] Bernardo, R. (2002). Breeding for quantitative traits in plants. Stemma Press.

[tpg270272-bib-0016] Bhat, M. A. , Kumar, V. , Bhat, M. A. , Wani, I. A. , Dar, F. L. , Farooq, I. , Bhatti, F. , Koser, R. , Rahman, S. , & Jan, A. T. (2020). Mechanistic insights of the interaction of plant growth‐promoting rhizobacteria (PGPR) with plant roots toward enhancing plant productivity by alleviating salinity stress. Frontiers in Microbiology, 11, 1952. 32973708 10.3389/fmicb.2020.01952 PMC7468593

[tpg270272-bib-0017] Blum, A. (2011). Drought resistance—Is it really a complex trait? Functional Plant Biology, 38(10), 753–757. 32480932 10.1071/FP11101

[tpg270272-bib-0018] Bradbury, P. J. , Zhang, Z. , Kroon, D. E. , Casstevens, T. M. , Ramdoss, Y. , & Buckler, E. S. (2007). TASSEL: Software for association mapping of complex traits in diverse samples. Bioinformatics, 23(19), 2633–2635. 17586829 10.1093/bioinformatics/btm308

[tpg270272-bib-0019] Burner, N. , Akiina, P. P. A. , Song, Q. , Harris, D. K. , & Li, Z. (2025). Identifying canopy wilting QTLs and evaluating remote sensing approaches for selecting drought tolerant soybean. Theoretical and Applied Genetics, 138, 276. 41087755 10.1007/s00122-025-05063-y PMC12521297

[tpg270272-bib-0020] Cabrera‐Bosquet, L. , Molero, G. , Nogués, S. , & Araus, J. L. (2009). Water and nitrogen conditions affect the relationships of Δ^13^C and Δ^18^O to gas exchange and growth in durum wheat. Journal of Experimental Botany, 60(6), 1633–1644. 19246596 10.1093/jxb/erp028 PMC2671614

[tpg270272-bib-0021] Chamarthi, S. K. , Kaler, A. S. , Abdel‐Haleem, H. , Fritschi, F. B. , Gillman, J. D. , Ray, J. D. , Smith, J. R. , Dhanapal, A. P. , King, C. A. , & Purcell, L. C. (2021). Identification and confirmation of loci associated with canopy wilting in soybean using genome‐wide association mapping. Frontiers in Plant Science, 12, 698116. 34335664 10.3389/fpls.2021.698116 PMC8317169

[tpg270272-bib-0022] Chamarthi, S. K. , Kaler, A. S. , Abdel‐Haleem, H. , Fritschi, F. B. , Gillman, J. D. , Ray, J. D. , Smith, J. R. , & Purcell, L. C. (2023). Identification of genomic regions associated with the plasticity of carbon 13 ratio in soybean. The Plant Genome, 16(1), e20284. 36411598 10.1002/tpg2.20284

[tpg270272-bib-0023] Chamarthi, S. K. , Purcell, L. C. , Fritschi, F. B. , Ray, J. D. , Smith, J. R. , Kaler, A. S. , King, C. A. , & Gillman, J. D. (2024). Association mapping for water use efficiency in soybean identifies previously reported and novel loci and permits genomic prediction. Frontiers in Plant Science, 15, 1486736. 39670270 10.3389/fpls.2024.1486736 PMC11634610

[tpg270272-bib-0024] Chapman, S. , Merz, T. , Chan, A. , Jackway, P. , Hrabar, S. , Dreccer, M. , Holland, E. , Zheng, B. , Ling, T. , & Jimenez‐Berni, J. (2014). Pheno‐copter: A low‐altitude, autonomous remote‐sensing robotic helicopter for high‐throughput field‐based phenotyping. Agronomy, 4(2), 279–301.

[tpg270272-bib-0025] Charlson, D. V. , Bhatnagar, S. , King, C. A. , Ray, J. D. , Sneller, C. H. , Carter, T. E. , & Purcell, L. C. (2009). Polygenic inheritance of canopy wilting in soybean [*Glycine max* (L.) Merr.]. Theoretical and Applied Genetics, 119(4), 587–594. 19471903 10.1007/s00122-009-1068-4

[tpg270272-bib-0026] Chen, D. , Neumann, K. , Friedel, S. , Kilian, B. , Chen, M. , Altmann, T. , & Klukas, C. (2014). Dissecting the phenotypic components of crop plant growth and drought responses based on high‐throughput image analysis. The Plant Cell, 26(12), 4636–4655. 25501589 10.1105/tpc.114.129601 PMC4311194

[tpg270272-bib-0027] Demicheli, J. , Sabljic, I. , Beguy, G. , Ploschuk, E. , Sahrawy, M. , Serrato, A. J. , & Pagano, E. A. (2023). Improving drought tolerance in soybean by classical breeding leads to physiological adjustments of photosynthesis and stomata functioning. Plant Stress, 10, 100275.

[tpg270272-bib-0028] Dhanapal, A. P. , Ray, J. D. , Singh, S. K. , Hoyos‐Villegas, V. , Smith, J. R. , Purcell, L. C. , King, C. A. , Cregan, P. B. , Song, Q. , & Fritschi, F. B. (2015). Genome‐wide association study (GWAS) of carbon isotope ratio (δ^13^C) in diverse soybean [*Glycine max* (L.) Merr.] genotypes. Theoretical and Applied Genetics, 128, 73–91. 25367378 10.1007/s00122-014-2413-9

[tpg270272-bib-0029] Dhanapal, A. P. , Ray, J. D. , Singh, S. K. , Hoyos‐Villegas, V. , Smith, J. R. , Purcell, L. C. , King, C. A. , & Fritschi, F. B. (2015). Genome‐wide association analysis of diverse soybean genotypes reveals novel markers for nitrogen traits. The Plant Genome, 8(3), plantgenome2014.2011.0086. 10.3835/plantgenome2014.11.0086 33228264

[tpg270272-bib-0030] Du, Y. , Zhao, Q. , Chen, L. , Yao, X. , & Xie, F. (2020). Effect of drought stress at reproductive stages on growth and nitrogen metabolism in soybean. Agronomy, 10(2), 302.

[tpg270272-bib-0031] Dubey, A. , Kumar, A. , Abd_Allah, E. F. , Hashem, A. , & Khan, M. L. (2019). Growing more with less: Breeding and developing drought resilient soybean to improve food security. Ecological Indicators, 105, 425–437.

[tpg270272-bib-0032] El Haddad, N. , Choukri, H. , Ghanem, M. E. , Smouni, A. , Mentag, R. , Rajendran, K. , Hejjaoui, K. , Maalouf, F. , & Kumar, S. (2021). High‐temperature and drought stress effects on growth, yield and nutritional quality with transpiration response to vapor pressure deficit in lentil. Plants, 11(1), 95. 35009098 10.3390/plants11010095 PMC8747359

[tpg270272-bib-0033] Falk, K. G. , Jubery, T. Z. , O'Rourke, J. A. , Singh, A. , Sarkar, S. , Ganapathysubramanian, B. , & Singh, A. K. (2020). Soybean root system architecture trait study through genotypic, phenotypic, and shape‐based clusters. Plant Phenomics, 2020, 1925495. 33313543 10.34133/2020/1925495 PMC7706349

[tpg270272-bib-0034] FAOSTAT . (2022). Crops and livestock products of the United Nations . https://openknowledge.fao.org/server/api/core/bitstreams/cd12276d-6933-4971-8fb9-b577c8bfad5c/content/cc2211en.html#chapter-2

[tpg270272-bib-0035] Farquhar, G. D. , Ehleringer, J. R. , & Hubick, K. T. (1989). Carbon isotope discrimination and photosynthesis. Annual Review of Plant Physiology and Plant Molecular Biology, 40, 503–537.

[tpg270272-bib-0036] Fehr, W. (1987). Principles of cultivar development. Volume 1: Theory and technique. Macmillian Publishing Company.

[tpg270272-bib-0037] Fuchs, M. , & Tanner, C. B. (1966). Infrared thermometry of vegetation. Agronomy Journal, 58(6), 597–601.

[tpg270272-bib-0038] Fukai, S. , & Mitchell, J. (2022). Role of canopy temperature depression in rice. Crop and Environment, 1(3), 198–213.

[tpg270272-bib-0039] Gates, D. M. (1968). Transpiration and leaf temperature. Annual Review of Plant Physiology, 19(1), 211–238.

[tpg270272-bib-0040] Gregorio Jorge, J. , Villalobos‐López, M. A. , Chavarría‐Alvarado, K. L. , Ríos‐Meléndez, S. , López‐Meyer, M. , & Arroyo‐Becerra, A. (2020). Genome‐wide transcriptional changes triggered by water deficit on a drought‐tolerant common bean cultivar. BMC Plant Biology, 20(1), Article 525. 33203368 10.1186/s12870-020-02664-1 PMC7672829

[tpg270272-bib-0041] Hall, D. , Tegstrom, C. , & Ingvarsson, P. K. (2010). Using association mapping to dissect the genetic basis of complex traits in plants. Briefings in Functional Genomics, 9(2), 157–165. 20053815 10.1093/bfgp/elp048

[tpg270272-bib-0042] Honna, P. T. , Fuganti‐Pagliarini, R. , Ferreira, L. C. , Molinari, M. D. C. , Marin, S. R. R. , De Oliveira, M. C. N. , Farias, J. R. B. , Neumaier, N. , Mertz‐Henning, L. M. , Kanamori, N. , Nakashima, K. , Takasaki, H. , Urano, K. , Shinozaki, K. , Yamaguchi‐Shinozaki, K. , Desidério, J. A. , & Nepomuceno, A. L. (2016). Molecular, physiological, and agronomical characterization, in greenhouse and in field conditions, of soybean plants genetically modified with AtGolS2 gene for drought tolerance. Molecular Breeding, 36(11), 157.

[tpg270272-bib-0043] Honsdorf, N. , March, T. J. , Berger, B. , Tester, M. , & Pillen, K. (2014). High‐throughput phenotyping to detect drought tolerance QTL in wild barley introgression lines. PLoS ONE, 9(5), e97047. 24823485 10.1371/journal.pone.0097047 PMC4019662

[tpg270272-bib-0044] Hou, M. , Tian, F. , Zhang, T. , & Huang, M. (2019). Evaluation of canopy temperature depression, transpiration, and canopy greenness in relation to yield of soybean at reproductive stage based on remote sensing imagery. Agricultural Water Management, 222, 182–192.

[tpg270272-bib-0045] Hwang, S. , King, C. A. , Chen, P. , Ray, J. D. , Cregan, P. B. , Carter, T. E. , Li, Z. , Abdel‐Haleem, H. , Matson, K. W. , Schapaugh, W. , & Purcell, L. C. (2016). Meta‐analysis to refine map position and reduce confidence intervals for delayed‐canopy‐wilting QTLs in soybean. Molecular Breeding, 36(7), 91.

[tpg270272-bib-0046] Hwang, S. , King, C. A. , Ray, J. D. , Cregan, P. B. , Chen, P. , Carter, T. E. , Li, Z. , Abdel‐Haleem, H. , Matson, K. W. , Schapaugh, W. , & Purcell, L. C. (2015). Confirmation of delayed canopy wilting QTLs from multiple soybean mapping populations. Theoretical and Applied Genetics, 128(10), 2047–2065. 26163767 10.1007/s00122-015-2566-1

[tpg270272-bib-0047] Jones, H. (2010). Remote detection of crop water “stress” and distinguishing it from other stresses. In XXVIII International Horticultural Congress on Science and Horticulture for People (IHC2010): International symposium on 922 (pp. 23–34). International Society for Horticulture.

[tpg270272-bib-0048] Jones, H. G. (2004). Application of thermal imaging and infrared sensing in plant physiology and ecophysiology. Advances in Botanical Research, 41, pp. 107–163.

[tpg270272-bib-0049] Jones, H. G. , Serraj, R. , Loveys, B. R. , Xiong, L. , Wheaton, A. , & Price, A. H. (2009). Thermal infrared imaging of crop canopies for the remote diagnosis and quantification of plant responses to water stress in the field. Functional Plant Biology, 36(11), 978–989. 32688709 10.1071/FP09123

[tpg270272-bib-0050] Jones, H. G. , & Vaughan, R. A. (2010). Remote sensing of vegetation: Principles, techniques, and applications. OUP Oxford.

[tpg270272-bib-0051] Kaler, A. S. , Abdel‐Haleem, H. , Fritschi, F. B. , Gillman, J. D. , Ray, J. D. , Smith, J. R. , & Purcell, L. C. (2020). Genome‐wide association mapping of dark green color index using a diverse panel of soybean accessions. Scientific Reports, 10(1), Article 5166. 32198467 10.1038/s41598-020-62034-7 PMC7083947

[tpg270272-bib-0052] Kaler, A. S. , Bazzer, S. K. , Sanz‐Saez, A. , Ray, J. D. , Fritschi, F. B. , & Purcell, L. C. (2018). Carbon isotope ratio fractionation among plant tissues of soybean. The Plant Phenome Journal, 1(1), 1–6.

[tpg270272-bib-0053] Kaler, A. S. , Dhanapal, A. P. , Ray, J. D. , King, C. A. , Fritschi, F. B. , & Purcell, L. C. (2017). Genome‐wide association mapping of carbon isotope and oxygen isotope ratios in diverse soybean genotypes. Crop Science, 57(6), 3085–3100.

[tpg270272-bib-0054] Kaler, A. S. , Gillman, J. D. , Beissinger, T. , & Purcell, L. C. (2019). Comparing different statistical models and multiple testing corrections for association mapping in soybean and maize. Frontiers in Plant Science, 10, 1794. 32158452 10.3389/fpls.2019.01794 PMC7052329

[tpg270272-bib-0055] Kaler, A. S. , & Purcell, L. C. (2019). Estimation of a significance threshold for genome‐wide association studies. BMC Genomics, 20(1), Article 618. 31357925 10.1186/s12864-019-5992-7 PMC6664749

[tpg270272-bib-0056] Kaler, A. S. , Ray, J. D. , Schapaugh, W. T. , Asebedo, A. R. , King, C. A. , Gbur, E. E. , & Purcell, L. C. (2018). Association mapping identifies loci for canopy temperature under drought in diverse soybean genotypes. Euphytica, 214(8), 135.

[tpg270272-bib-0057] Kaler, A. S. , Ray, J. D. , Schapaugh, W. T. , Davies, M. K. , King, C. A. , & Purcell, L. C. (2018). Association mapping identifies loci for canopy coverage in diverse soybean genotypes. Molecular Breeding, 38(5), 50.

[tpg270272-bib-0058] Kaler, A. S. , Ray, J. D. , Schapaugh, W. T. , King, C. A. , & Purcell, L. C. (2017). Genome‐wide association mapping of canopy wilting in diverse soybean genotypes. Theoretical and Applied Genetics, 130(10), 2203–2217. 28730464 10.1007/s00122-017-2951-z

[tpg270272-bib-0059] Kapoor, D. , Bhardwaj, S. , Landi, M. , Sharma, A. , Ramakrishnan, M. , & Sharma, A. (2020). The impact of drought in plant metabolism: How to exploit tolerance mechanisms to increase crop production. Applied Sciences, 10(16), 5692.

[tpg270272-bib-0060] Kumar, M. , Govindasamy, V. , Rane, J. , Singh, A. K. , Choudhary, R. L. , Raina, S. K. , George, P. , Aher, L. K. , & Singh, N. P. (2017). Canopy temperature depression (CTD) and canopy greenness associated with variation in seed yield of soybean genotypes grown in semi‐arid environment. South African Journal of Botany, 113, 230–238.

[tpg270272-bib-0061] Leinonen, I. , Grant, O. M. , Tagliavia, C. P. P. , Chaves, M. M. , & Jones, H. G. (2006). Estimating stomatal conductance with thermal imagery. Plant, Cell & Environment, 29(8), 1508–1518. 10.1111/j.1365-3040.2006.01528.x 16898014

[tpg270272-bib-0062] Lepekhov, S. B. (2022). Canopy temperature depression for drought‐ and heat stress tolerance in wheat breeding. Vavilovskii Zhurnal Genetiki i Selektsii, 26(2), 196–201. 35434483 10.18699/VJGB-22-24 PMC8983952

[tpg270272-bib-0063] Li, S. , Cao, Y. , Wang, C. , Yan, C. , Sun, X. , Zhang, L. , Wang, W. , & Song, S. (2023). Genome wide association mapping for yield related traits in soybean (*Glycine max*) under well watered and drought stressed conditions. Frontiers in Plant Science, 14, 1265574. 37877078 10.3389/fpls.2023.1265574 PMC10593458

[tpg270272-bib-0064] Li, S. , Wang, C. , Yan, C. , Sun, X. , Zhang, L. , Cao, Y. , Wang, W. , & Song, S. (2024). Identification and fine mapping of qSW2 for leaf slow wilting in soybean. The Crop Journal, 12(1), 244–251.

[tpg270272-bib-0065] Lim, C. W. , Baek, W. , Jung, J. , Kim, J.‐H. , & Lee, S. C. (2015). Function of ABA in stomatal defense against biotic and drought stresses. International Journal of Molecular Sciences, 16(7), 15251–15270. 26154766 10.3390/ijms160715251 PMC4519898

[tpg270272-bib-0066] Liu, X. , Huang, M. , Fan, B. , Buckler, E. S. , & Zhang, Z. (2016). Iterative usage of fixed and random effect models for powerful and efficient genome‐wide association studies. PLoS Genetics, 12(2), e1005767. 26828793 10.1371/journal.pgen.1005767 PMC4734661

[tpg270272-bib-0067] LMC International . (2023). The economic impact of U.S. soybeans & end products on the U.S. economy . https://www.nopa.org/wp-content/uploads/2023/08/0LMC_SoyEconStudy_Aug2023.pdf

[tpg270272-bib-0068] Lopes, M. S. , & Reynolds, M. P. (2010). Partitioning of assimilates to deeper roots is associated with cooler canopies and increased yield under drought in wheat. Functional Plant Biology, 37(2), 147–156.

[tpg270272-bib-0069] Maes, W. H. , & Steppe, K. (2012). Estimating evapotranspiration and drought stress with ground‐based thermal remote sensing in agriculture: A review. Journal of Experimental Botany, 63(13), 4671–4712. 22922637 10.1093/jxb/ers165

[tpg270272-bib-0070] Mason, R. E. , Hays, D. B. , Mondal, S. , Ibrahim, A. M. , & Basnet, B. R. (2013). QTL for yield, yield components and canopy temperature depression in wheat under late sown field conditions. Euphytica, 194, 243–259.

[tpg270272-bib-0071] Menke, E. , Steketee, C. J. , Song, Q. , Schapaugh, W. T. , Carter, T. E. , Fallen, B. , & Li, Z. (2024). Genetic mapping reveals the complex genetic architecture controlling slow canopy wilting in soybean. Theoretical and Applied Genetics, 137(5), 107. 38632129 10.1007/s00122-024-04609-w PMC11024021

[tpg270272-bib-0072] Money, D. , Gardner, K. , Migicovsky, Z. , Schwaninger, H. , Zhong, G.‐Y. , & Myles, S. (2015). LinkImpute: Fast and accurate genotype imputation for nonmodel organisms. G3 Genes|Genomes|Genetics, 5(11), 2383–2390. 26377960 10.1534/g3.115.021667 PMC4632058

[tpg270272-bib-0073] Monnens, D. , Denison, R. F. , & Sadok, W. (2023). Rising vapor‐pressure deficit increases nitrogen fixation in a legume crop. New Phytologist, 239(1), 54–65. 37097254 10.1111/nph.18929

[tpg270272-bib-0074] Ninanya, J. , Ramírez, D. A. , Rinza, J. , Silva‐Díaz, C. , Cervantes, M. , García, J. , & Quiroz, R. (2021). Canopy temperature as a key physiological trait to improve yield prediction under water restrictions in potato. Agronomy, 11(7), 1436.

[tpg270272-bib-0075] Oguz, M. C. , Aycan, M. , Oguz, E. , Poyraz, I. , & Yildiz, M. (2022). Drought stress tolerance in plants: Interplay of molecular, biochemical and physiological responses in important development stages. Physiologia, 2(4), 180–197.

[tpg270272-bib-0076] Prince, S. J. , Beena, R. , Gomez, S. M. , Senthivel, S. , & Babu, R. C. (2015). Mapping consistent rice (*Oryza sativa* L.) yield QTLs under drought stress in target rainfed environments. Rice, 8, Article 25. 26206756 10.1186/s12284-015-0053-6 PMC4513014

[tpg270272-bib-0077] Purcell, L. C. , Edwards, J. T. , & Brye, K. R. (2007). Soybean yield and biomass responses to cumulative transpiration: Questioning widely held beliefs. Field Crops Research, 101(1), 10–18.

[tpg270272-bib-0078] Purcell, L. C. , & Specht, J. E. (2004). Physiological traits for ameliorating drought stress. In Soybeans: Improvement, production, and uses (Vol. 16, pp. 569–620). American Society of Agronomy, Crop Science Society of America, and the Soil Science Society of America.

[tpg270272-bib-0079] Purushothaman, R. , Thudi, M. , Krishnamurthy, L. , Upadhyaya, H. D. , Kashiwagi, J. , Gowda, C. L. L. , & Varshney, R. K. (2015). Association of mid‐reproductive stage canopy temperature depression with the molecular markers and grain yields of chickpea (*Cicer arietinum* L.) germplasm under terminal drought. Field Crops Research, 174, 1–11.

[tpg270272-bib-0080] Quinlan, A. R. , & Hall, I. M. (2010). BEDTools: A flexible suite of utilities for comparing genomic features. Bioinformatics, 26(6), 841–842. 20110278 10.1093/bioinformatics/btq033 PMC2832824

[tpg270272-bib-0081] Revelle, W. R. (2017). psych: Procedures for personality and psychological research . https://revelle.r-universe.dev/psych

[tpg270272-bib-0082] Reynolds, M. , Dreccer, F. , & Trethowan, R. (2007). Drought‐adaptive traits derived from wheat wild relatives and landraces. Journal of experimental botany, 58(2), 177–186. 17185737 10.1093/jxb/erl250

[tpg270272-bib-0083] Reynolds, M. , Manes, Y. , Izanloo, A. , & Langridge, P. (2009). Phenotyping approaches for physiological breeding and gene discovery in wheat. Annals of Applied Biology, 155(3), 309–320.

[tpg270272-bib-0084] Sah, S. K. , Reddy, K. R. , & Li, J. (2016). Abscisic acid and abiotic stress tolerance in crop plants. Frontiers in Plant Science, 7, 571. 27200044 10.3389/fpls.2016.00571 PMC4855980

[tpg270272-bib-0085] Saleem, A. , Roldán‐Ruiz, I. , Aper, J. , & Muylle, H. (2022). Genetic control of tolerance to drought stress in soybean. BMC Plant Biology, 22(1), Article 615. 36575367 10.1186/s12870-022-03996-w PMC9795773

[tpg270272-bib-0086] Salehi‐Lisar, S. Y. , & Bakhshayeshan‐Agdam, H. (2020). Agronomic crop responses and tolerance to drought stress. In M. Hasanuzzaman (Ed.), Agronomic crops: Volume 3: Stress responses and tolerance (pp. 63–91). Springer.

[tpg270272-bib-0087] SAS Institute Inc . (2023). The GLIMMIX procedure. In SAS/STAT® 15.3 user's guide. SAS Institute Inc.

[tpg270272-bib-0088] Schmutz, J. , Cannon, S. B. , Schlueter, J. , Ma, J. , Mitros, T. , Nelson, W. , Hyten, D. L. , Song, Q. , Thelen, J. J. , Cheng, J. , Xu, D. , Hellsten, U. , May, G. D. , Yu, Y. , Sakurai, T. , Umezawa, T. , Bhattacharyya, M. K. , Sandhu, D. , Valliyodan, B. , … Jackson, S. A. (2010). Genome sequence of the palaeopolyploid soybean. Nature, 463(7278), 178–183. 20075913 10.1038/nature08670

[tpg270272-bib-0089] Seleiman, M. F. , Al‐Suhaibani, N. , Ali, N. , Akmal, M. , Alotaibi, M. , Refay, Y. , Dindaroglu, T. , Abdul‐Wajid, H. H. , & Battaglia, M. L. (2021). Drought stress impacts on plants and different approaches to alleviate its adverse effects. Plants, 10(2), 259. 33525688 10.3390/plants10020259 PMC7911879

[tpg270272-bib-0090] Seversike, T. M. , Sermons, S. M. , Sinclair, T. R. , Carter, T. E., Jr. , & Rufty, T. W. (2013). Temperature interactions with transpiration response to vapor pressure deficit among cultivated and wild soybean genotypes. Physiologia Plantarum, 148(1), 62–73. 22989317 10.1111/j.1399-3054.2012.01693.x

[tpg270272-bib-0091] Sinclair, T. R. , Messina, C. D. , Beatty, A. , & Samples, M. (2010). Assessment across the United States of the benefits of altered soybean drought traits. Agronomy Journal, 102(2), 475–482.

[tpg270272-bib-0092] Singh, P. , & Kanemasu, E. (1983). Leaf and canopy temperatures of pearl millet genotypes under irrigated and nonirrigated conditions. Agronomy Journal, 75(3), 497–501.

[tpg270272-bib-0093] Song, Q. , Hyten, D. L. , Jia, G. , Quigley, C. V. , Fickus, E. W. , Nelson, R. L. , & Cregan, P. B. (2013). Development and evaluation of SoySNP50K, a high‐density genotyping array for soybean. PLoS ONE, 8(1), e54985. 23372807 10.1371/journal.pone.0054985 PMC3555945

[tpg270272-bib-0094] Song, Q. , Hyten, D. L. , Jia, G. , Quigley, C. V. , Fickus, E. W. , Nelson, R. L. , & Cregan, P. B. (2015). Fingerprinting soybean germplasm and its utility in genomic research. G3 Genes|Genomes|Genetics, 5(10), 1999–2006. 26224783 10.1534/g3.115.019000 PMC4592982

[tpg270272-bib-0095] Steketee, C. J. , Schapaugh, W. T. , Carter, T. E., Jr. , & Li, Z. (2020). Genome‐wide association analyses reveal genomic regions controlling canopy wilting in soybean. G3 Genes|Genomes|Genetics, 10(4), 1413–1425. 32111650 10.1534/g3.119.401016 PMC7144087

[tpg270272-bib-0096] Steketee, C. J. , Sinclair, T. R. , Riar, M. K. , Schapaugh, W. T. , & Li, Z. (2019). Unraveling the genetic architecture for carbon and nitrogen related traits and leaf hydraulic conductance in soybean using genome‐wide association analyses. BMC Genomics, 20(1), Article 811. 31694528 10.1186/s12864-019-6170-7 PMC6836393

[tpg270272-bib-0097] Thompson, A. L. , Thorp, K. R. , Conley, M. M. , Roybal, M. , Moller, D. , & Long, J. C. (2020). A data workflow to support plant breeding decisions from a terrestrial field‐based high‐throughput plant phenotyping system. Plant Methods, 16(1), Article 97. 32695214 10.1186/s13007-020-00639-9 PMC7364621

[tpg270272-bib-0098] Van der Laan, L. , de Azevedo Peixoto, L. , & Singh, A. K. (2025). Genetic dissection of heat stress tolerance in soybean through genome‐wide association studies and use of genomic prediction to enhance breeding applications. npj Science of Plants, 1, Article 9.

[tpg270272-bib-0099] Wang, J. , Tang, Y. , & Zhang, Z. (2022). Performing genome‐wide association studies with multiple models using GAPIT. In Genome‐wide association studies (pp. 199–217). Springer. 10.1007/978-1-0716-2237-7_13 35641767

[tpg270272-bib-0100] Wang, W. , Wang, C. , Pan , D. , Zhang , Y. , Luo , B. , & Ji , J. (2018). Effects of drought stress on photosynthesis and chlorophyll fluorescence images of soybean (*Glycine max*) seedlings. International Journal of Agricultural and Biological Engineering, 11(2), 196–201.

[tpg270272-bib-0101] Xu, C. , He, Y. , Sun, S. , Song, W. , Wu, T. , Han, T. , & Wu, C. (2020). Analysis of soybean yield formation differences across different production regions in China. Agronomy Journal, 112(5), 4195–4206.

[tpg270272-bib-0102] Ye, H. , Song, L. , Schapaugh, W. T. , Ali, M. D. L. , Sinclair, T. R. , Riar, M. K. , Mutava, R. N. , Li, Y. , Vuong, T. , Valliyodan, B. , Pizolato Neto, A. , Klepadlo, M. , Song, Q. , Shannon, J. G. , Chen, P. , & Nguyen, H. T. (2020). The importance of slow canopy wilting in drought tolerance in soybean. Journal of Experimental Botany, 71(2), 642–652. 30980084 10.1093/jxb/erz150 PMC6946001

[tpg270272-bib-0103] Zhang, Q. , Li, J. , Zhang, W. , Yan, S. , Wang, R. , Zhao, J. , Li, Y. , Qi, Z. , Sun, Z. , & Zhu, Z. (2012). The putative auxin efflux carrier OsPIN3t is involved in the drought stress response and drought tolerance. The Plant Journal, 72(5), 805–816. 22882529 10.1111/j.1365-313X.2012.05121.x

